# An interorgan neuroimmune circuit promotes visceral hypersensitivity

**DOI:** 10.21203/rs.3.rs-6221928/v1

**Published:** 2025-03-17

**Authors:** Brian Kim, Zhen Wang, Xia Meng, Zili Xie, Chia Chun Hor, wenwen zhang, Xu Li, Keaton Song, Kelsey Auyeung, Hisato Iriki, Rintaro Shibuya, Wen Zhang, Jack Major, Lydia Zamidar, Daniel Yassky, Olivia Seker, Joshua Wollam, Christiane Villescaz, Alan Vest, Swetha Srinivasan, Michelle Solomon, Rui Chang, Shruti Naik, Masato Kubo, Veena Viswanath, Shawn Xu, Xinzhong Dong, David Artis, Bo Duan, Hongzhen Hu

**Affiliations:** Icahn School of Medicine at Mount Sinai; Icahn School of Medicine at Mount Sinai; Icahn School of Medicine at Mount Sinai; Icahn School of Medicine at Mount Sinai; University of Michigan-Ann Arbor; University of Michigan-Ann Arbor; Icahn School of Medicine at Mount Sinai; Icahn School of Medicine at Mount Sinai; Icahn School of Medicine at Mount Sinai; Icahn School of Medicine at Mount Sinai; Icahn School of Medicine at Mount Sinai; Weill Cornell Medical College, Cornell University; Icahn School of Medicine at Mount Sinai; Icahn School of Medicine at Mount Sinai; Icahn School of Medicine at Mount Sinai; Icahn School of Medicine at Mount Sinai; Escient Pharmaceuticals; Escient Pharmaceuticals; Escient Pharmaceuticals; Escient Pharmaceuticals; Escient Pharmaceuticals; Yale University School of Medicine; Department of Immunology and Immunotherapy, Icahn School of Medicine at Mt Sinai; RIKEN; Escient Pharmaceuticals; University of Michigan-Ann Arbor; The Solomon H. Snyder Department of Neuroscience, Center for Sensory Biology, Johns Hopkins University School of Medicine, Baltimore, MD 21205; Weill Cornell Medical College, Cornell University; University of Michigan; Icahn School of Medicine at Mount Sinai

## Abstract

Visceral pain disorders such as interstitial cystitis/bladder pain syndrome (IC/BPS) and irritable bowel syndrome (IBS) often manifest concurrently in the bladder and colon. Yet, the mechanistic basis of such comorbidities and the transmission of neural hypersensitivity across organ systems has remained a mystery. Here, we identify a mast cell-sensory neuron circuit that initiates bladder inflammation and simultaneously propagates neural hypersensitivity to the colon in a murine model of IC/BPS. We unveil anatomic heterogeneity of mast cells in relation to nociceptors in the bladder and their critical dependence on Mas-related G protein-coupled receptor B2 (MrgprB2) to promote visceral hypersensitivity. Employing retrograde neuronal tracing, in vivo calcium imaging, and intersectional genetics, we uncover a population of polyorganic sensory neurons that simultaneously innervate multiple organs and exhibit functional convergence. Importantly, using humanized mice, we demonstrate that pharmacological blockade of mast cell-expressed MRGPRX2, the human ortholog of MrgprB2, attenuates both bladder pathology and colonic hypersensitivity. Our studies reveal evolutionarily conserved neuroimmune mechanisms by which immune cells can directly convey signals from one organ to another through sensory neurons, in the absence of physical proximity, representing a new therapeutic paradigm.

Crosstalk between organs is traditionally attributed to humoral factors that circulate throughout the body^6^. Moreover, physiology across multiple organs is coordinated centrally by the brain by integrating sensory inputs and motor outputs^7^. Notwithstanding these systemic mechanisms, it has been hypothesized for decades that peripheral organs such as the bladder and gut can interact locally and directly^8^. However, the mechanisms that could account for such interorgan communications remain uncharted.

The bladder and colon are primarily expulsive organs designed to remove metabolic waste, irritants, and pathogens. To optimize their excretory motor functions, both organs must sense a variety of mechanical and immunological cues^9,10^. However, in the setting of pathologic conditions like inflammation, homeostatic sensory signals become aberrant and drive detrimental visceral pain. Further, inflammation in one organ can induce widespread visceral hypersensitivity^1–4^. IC/BPS is a chronic and debilitating condition that manifests as pain, burning, and urinary urgency, and affects approximately 5% of women^11,12^. In addition to having limited treatments, IC/BPS is strongly associated with the development of comorbid visceral pain conditions such as IBS^13^. Notably, 30–75% of patients with IC/BPS report symptoms resembling IBS^14^, indicating that there is a unifying pathophysiology.

While it is increasingly appreciated that immune cells and cytokines can directly activate sensory neurons to facilitate crosstalk between the brain and peripheral organs^7,15^, whether neuroimmune interactions underlie direct communication between peripheral organs remains poorly defined. A homeostatic interorgan neural reflex has long been proposed between the bladder and the colon^16^, yet the role of this bladder-colon circuit in disease remains poorly understood. We hypothesized that interorgan neuroimmune networks may transmit irritability triggered by inflammation from the bladder to the colon.

## Bladder inflammation promotes intestinal irritability

**Figure 1:**
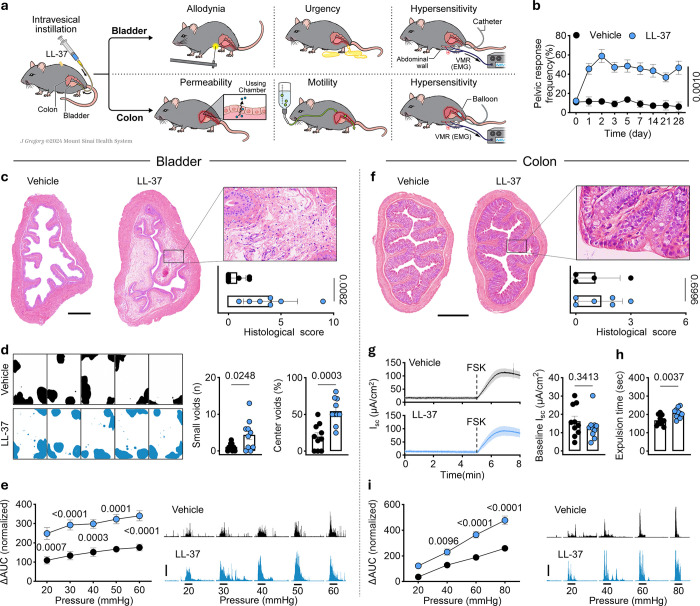
Bladder inflammation induces colon hypersensitivity. **a**, Schematic of the experimental protocol for murine IC/BPS-like disease induction followed by behavioral and functional assays targeting the bladder or colon. **b**, Response frequencies to suprapubic mechanical stimuli by a 0.04 g von Frey filament of mice treated with vehicle (n = 11) or LL-37 (n = 14). **c**, Representative bladder histology (left) and histological scores (right) at day 3 post-instillation in vehicle- or LL-37-treated mice (n = 7 per group). Box indicates representative edema and infiltration of leukocytes including PMNs. Scale bar, 500 μm. **d**, Urination patterns (left), small void numbers (middle), and center void percentages (right) of vehicle and LL-37-treated mice (n = 10 per group). **e**, VMR to urinary bladder distention as measured by the area under the curve (AUC) in mice treated intravesically with vehicle (n = 8) or LL-37 (n = 11); normalized to baseline (see Methods). Biological replicates are indicated in graphs (left), and representative traces are shown on the right. Scale bars, 300 μV (vertical) and 10 s (horizontal). **f**, Representative distal colon histology (left) and histological scores (right) at day 3 post-instillation in vehicle- or LL-37-treated mice (n = 4 and 6, respectively). Scale bar, 500 μm. **g**, Average transepithelial currents (I_SC_; left) and quantification of baseline I_SC_ (right) recorded in distal colonic mucosa obtained from vehicle- or LL-37-treated mice (n = 10 per group). **h**, Expulsion time following the insertion of a 3 mm glass bead into the colon of vehicle- or LL-37-treated mice (n = 10 per group). **i**, VMR to colorectal distention in vehicle- or LL-37-treated mice (n = 8 per group). Biological replicates are indicated in graphs (left), and representative traces are shown on the right. Scale bars, 300 μV (vertical) and 10 s (horizontal). Data are presented as mean ± s.e.m. *P* values are shown in plots. **b**, **e**, **i**, Two-way repeated ANOVA with Sidak’s multiple comparisons test. **c, d, f, g**, **h**, Two-sided Student’s t-tests with Welch’s correction. The diagram in **a** was created by J. Gregory.

We first examined how bladder inflammation can trigger local visceral hypersensitivity using an established model of IC/BPS^5^. To this end, we delivered LL-37, an antimicrobial peptide associated with human IC/BPS^17^, intravesically to the bladder of mice to elicit pain and inflammation (Fig. 1a). Indeed, we found that LL-37 exposure consistently induced chronic pelvic pain, or allodynia, compared to vehicle control (Fig. 1b). Acute inflammatory pathology in the bladder was reflected by severe edema and infiltration of polymorphonuclear leukocytes (PMNs) in the lamina propria of mice exposed to LL-37 (Fig. 1c). Bladder motor dysfunction was demonstrated by urinary urgency behavior (Fig. 1d). Moreover, electromyogram (EMG) recordings revealed that visceromotor responses (VMR) to graded urinary bladder distension (UBD) were enhanced in LL-37-treated mice, which was indicative of bladder hypersensitivity (Fig. 1e).

Given that patients with IC/BPS are known to have a significantly increased risk for both inflammatory and irritable bowel diseases^13,18^, we next reasoned that local bladder inflammation is sufficient to drive colonic pathology in this model. However, despite experiencing robust bladder inflammation (Fig. 1c), mice did not exhibit any evidence of inflammatory pathology in the colon (Fig. 1f). Further, no change in mucosal permeability was observed (Fig. 1g). We therefore characterized sensory and motor responses specifically in the colon. Notably, even in the absence of colon inflammation, IC/BPS-like disease resulted in the development of a constipation phenotype as evidenced by prolonged colonic glass bead expulsion time (Fig. 1h). Moreover, mice with IC/BPS-like symptoms exhibited marked colon irritability as shown by increased VMR to colorectal distension (CRD) (Fig. 1i). Together, these findings confirm that bladder inflammation can propagate neural hypersensitivity from the bladder to the colon, resulting in spontaneous IBS-like symptoms.

## Polyorganic sensory neurons drive interorgan irritability

**Figure 2:**
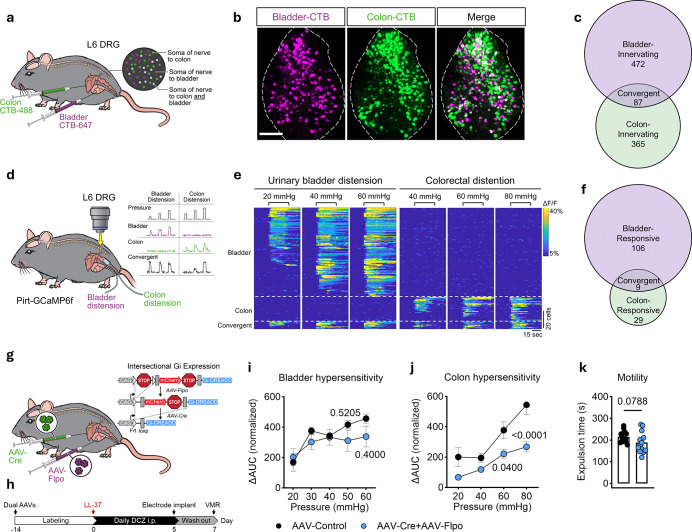
Polyorganic sensory neurons promote bladder-colon sensitization. **a**, Schematic of dual-color CTB retrograde labeling of DRG sensory neurons innervating the urinary bladder (CTB-647, purple) and the distal colon (CTB-488, green). **b**, Representative whole-mount images of DRG (L6) after tracing. Scale bar, 200 μm. **c**, Venn diagram of the numbers of L6 DRG sensory neurons with single (purple or green) or dual (gray) retrograde labeling from the bladder and distal colon of mice (n = 3). **d**, Schematic of *in vivo* calcium imaging of L6 DRG neurons. Graded mechanical stimulation of the bladder and colon were performed through an intravesical catheter or intracolonic balloon, respectively. The insets represent calcium traces from individual neurons and are shown and color coded according to the categories shown in (**e**). **e**, Heatmap of calcium responses of L6 DRG neurons obtained after sequential bladder-colon graded stimulation of *Pirt*^*Cre*^*;GCaMP6f*^*flox*/+^ mice. Neurons were functionally classified in three categories based on their response to stimuli and sorted by Δ*F/F*: Bladder (bladder responsive only), Colon (colon responsive only), and Convergent (dual bladder and colon responsive). Stimulations are shown on top of the heatmap. Scale bars, 20 cells (vertical) and 15 s (horizontal). **f**, Quantification of recorded cells per category shown on (**e**), purple: bladder responsive only, green: colon responsive only, and gray: dual bladder-colon responsive neurons. N = 144 cells; from 4 mice. **g**, Intersectional genetic strategy to selectively express an inhibitory GPCR (Gi-DREADD) in polyorganic sensory neurons using a microinjection of AAV9-hSyn-Flpo into the bladder and AAV9-hSyn-Cre into the colon of the *RC::FPGi* dual-recombinase responsive allele. The insets represent Flpo recombinase resulting in mCherry fluorescence, and further exposure to Cre recombinase resulting in Gi-DREADD expression in the overlapping populations. **h**, Timeline of administration of the Gi-DREADD ligand DCZ (100 μg kg^−1^), twice a day) for 5 d followed by a 2 d washout. **i**, **j**, **k**, VMR to bladder distension (**i**; n = 4 per group), colon distension (**j**; n = 10 AAV-Control, 9 AAV-Cre+AAV-Flpo), and colonic motility (**k**; n = 14 AAV-Control, 13 AAV-Cre+AAV-Flpo) in mice whose polyorganic sensory neurons were inhibited compared to controls following DCZ treatment upon IC/BPS induction. Data are presented as mean ± s.e.m. *P* values are shown in plots. **i**, **j**, Two-way repeated ANOVA with Sidak’s multiple comparisons test. **k**, Two-sided Student’s t-tests with Welch’s correction. The diagrams in **a**, **d**, **g** were created by J. Gregory.

The ensuing symptoms of interorgan colonic hypersensitivity, triggered by bladder inflammation without primary colon pathology, provoked the hypothesis that there is a direct bladder-colon neural circuit. To map peripheral neurons innervating the bladder and colon, we performed retrograde tracing from both organs using cholera toxin B (CTB) conjugated to two distinct fluorophores (Fig. 2a). Our results showed that the bladder was primarily innervated by sensory neurons arising from T13–L1/L5–S1 thoracolumbar sacral dorsal root ganglia (DRG) segments, with no input detected from vagal sensory neurons within the nodose ganglia (**Extended Data Fig. 1a, b**). By contrast, the distal colon, while also being largely innervated by the same DRG segments, demonstrated sparse innervation from the nodose ganglia (**Extended Data Fig. 1a, b**). Although the majority of sensory neurons were bladder- or colon-specific, notably, approximately 15.5% of L6 DRG neurons exhibited dual fluorophore labeling (Fig. 2b, c **and Extended Data Fig. 1b, c and Supplementary Video 1**). Additionally, the bladder and colon are innervated by two non-overlapping autonomic populations in major pelvic ganglia (**Extended Data Fig. 1a, c**), indicating that a significant proportion of spinal sensory neurons, rather than autonomic neurons, simultaneously innervate both the bladder and colon. To determine whether this shared innervation across organs reflects functional connectivity, we undertook *in vivo* calcium imaging of *Pirt*^*Cre*^;*GCaMP6f*^*flox*/+^ mice^19^ (Fig. 2d). Indeed, 7.8% of sensory neurons that responded to bladder distention could also be activated by distension of the colon alone (Fig. 2e, f **and Extended Data Fig. 1d, e**). Together, these data show that the bladder and colon are innervated by a shared population of functionally responsive sensory neurons. We define these populations as polyorganic sensory neurons.

We next reasoned that inhibition of polyorganic sensory neurons could suppress the propagation of irritability from the bladder to colon. To this end, we performed retrograde viral delivery in conjunction with intersectional genetic manipulation^20^ to functionally target this sensory neuron cluster (Fig. 2g). First, we injected AAV9-Flpo or control AAV9-mCherry into the bladder wall of *RC::FPDi* dual-Gi DREADD mice. Second, we injected AAV9-Cre or control AAV9-EGFP into the colon wall of these same mice (Fig. 2g). All injected viruses reach the thoracolumbar and lumbosacral DRG through retrograde transport. However, only convergent neurons that are co-infected with both AAV9-Flpo and AAV9-Cre express hM4Di, a chemogenetically activated inhibitory GPCR in dual-Gi DREADD mice (Fig. 2g). In other words, only polyorganic sensory neurons are capable of being chemogenetically inhibited in response to deschloroclozapine (DCZ). Mice were then treated with DCZ daily for 5 days (inhibition period) followed by a 2-day washout period (Fig. 2h). Upon induction of bladder inflammation, despite there being no difference in bladder hypersensitivity (Fig. 2i), chemogenetic inhibition of neurons co-innervating the bladder and colon resulted in attenuation of colonic hypersensitivity (Fig. 2j) and dysmotility (Fig. 2k) compared to controls. Collectively, these findings reveal that such polyorganic sensory neurons drive the development of interorgan visceral hypersensitivity along a direct bladder-colon neural axis.

## Mast cells and MrgprB2 trigger interorgan sensitization

**Figure 3:**
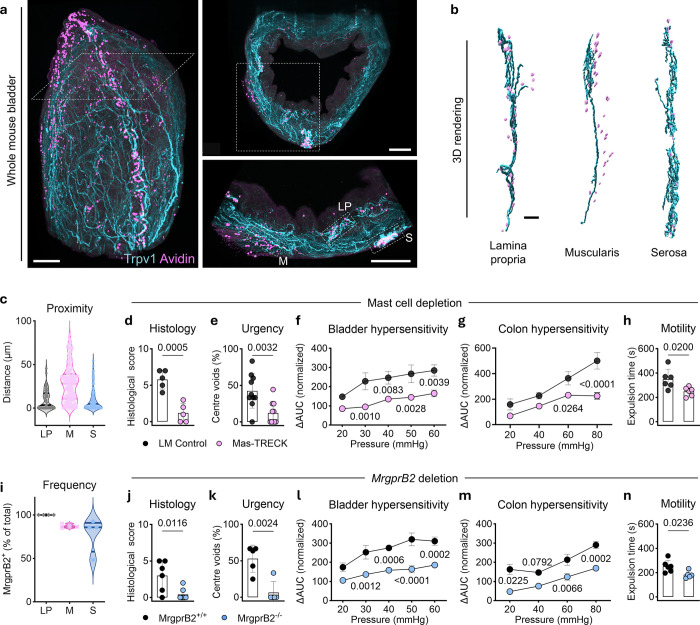
The bladder-colon hypersensitivity axis is mast cell- and MrgprB2-dependent. **a**, Left: Light sheet image of an optically cleared whole-mount bladder from *Trpv1*^*Cre*^*;Ai14*^*flox/flox*^ mice stained with the mast cell indicator Avidin. Top right: Selective slice views showing the projection of Trpv1^+^ spinal afferents and the distribution of Avidin^+^ mast cells across the lumen-surface axis of the bladder. Bottom right: Representative slice views showing anatomical proximity between mast cells and nociceptors highlighted by white boxes as lamina propria (LP), muscularis (M), and serosa (S). Scale bars, 500 μm. **b**, IMARIS 3D rendering of Avidin^+^ mast cells forming physical contact with Trpv1^+^ nociceptor fibers in the LP and S layers, but only being in slight proximity to fibers in the M layers of the bladder. Scale bar, 100 μm. **c**, IMARIS automated computational analysis of the minimum distance (μm) between modeled Avidin^+^ mast cells and modeled Trpv1^+^ nociceptor fibers across 3 histological layers in the bladder. LP (n = 192 cells), M (n = 235 cells), and S (n = 216 cells); from 4 mice. **d**, **e**, **f**, **g**, **h**, Evaluation of bladder pathology (**d**; n = 5 per group), urinary urgency (**e**; n = 10 per group), bladder hypersensitivity (**f**; n = 8 and 10, respectively), colonic hypersensitivity (**g**; n = 6 and 8, respectively), and motility (**h**; n = 6 per group) in intravesically LL-37-challenged and diphtheria toxin-injected littermates (LM) controls and Mas-TRECK mice. **i**, Percentages of tdTomato (MrgprB2^+^) Avidin^+^ cells in all Avidin^+^ mast cells in the LP (n = 393 cells), M (n = 133 cells), and S (n = 222 cells) layers of the bladder in *MrgprB2*^*Cre*^*;Ai9*^*flox/flox*^ mice (n = 4). **j**, **k**, **l**, **m**, **n**, Evaluations in bladder pathology (**j**; n = 6 and 7, respectively), urgency (**k**; n = 5 per group), bladder hypersensitivity (**l**; n = 6 and 7, respectively), colonic hypersensitivity (**m**; n = 6 per group), and motility (**n**; n = 6 and 5, respectively) in intravesically LL-37-challenged wild type (*MrgprB2*^+/+^) controls and MrgprB2 mutant mice (*MrgprB2*^−/−^). Data are presented as mean ± s.e.m. *P* values are shown in plots. **f**, **g**, **l**, **m**, Two-way repeated ANOVA with Sidak’s multiple comparisons test. **d**, **e**, **h**, **j**, **k**, **n**, Two-sided Student’s t-tests with Welch’s correction.

Tissue-resident mast cells are well known to relay nociceptive signals to sensory neurons across multiple barrier surfaces^21^. To visualize and quantify the spatial organization of mast cells in relation to nociceptors in the bladder, we performed whole-mount clearing and volumetric imaging of bladders from *Trpv1*^*Cre*^*;Ai14*^*flox/flox*^ tdTomato reporter mice in conjunction with Avidin staining to identify mast cells (Fig. 3a **and Supplementary Video 2**). While mast cells in the muscularis exhibited limited physical relationship to TRPV1^+^ sensory neurons, the majority of mast cells in the lamina propria formed physical contact with or were in close proximity to nociceptors (Fig. 3b, c **and Supplementary Video 3**), where IC/BPS-like inflammation was observed (Fig. 1c). To determine the role of mast cells in the bladder-colon hypersensitivity axis, we employed Mas-TRECK mice^22^, in which mast cells are conditionally depleted upon diphtheria toxin (DT) treatment. Notably, upon efficient depletion of bladder mast cells (**Extended Data Fig. 2a–c**), Mas-TRECK mice displayed attenuation of all the key pathologic and symptomatic signs of IC/BPS-like disease including decreased bladder inflammation (Fig. 3d), reduced urinary urgency (Fig. 3e), and diminished bladder hypersensitivity (Fig. 3f). Importantly, colon irritability (Fig. 3g) and dysmotility (Fig. 3h) were also alleviated, suggesting that mast cells are necessary to induce interorgan sensitization.

Beyond histamine, leukotriene C4 (LTC4) has recently been implicated in mast cell-neuron interactions that evoke both itch and even food avoidance behavior^23–26^. LTC4 triggers neurologic reflexes through its receptor CysLTR2. Thus, we sought to test whether pharmacological inhibition of CysLTR2 can treat visceral hypersensitivity using the well-established antagonist HAMI3379. We found that mice treated with HAMI3379 exhibited decreased bladder hypersensitivity in the setting of IC/BPS-like disease (**Extended Data Fig. 3a**). Moreover, colon hypersensitivity was also significantly attenuated (**Extended Data Fig. 3b**). Taken together, these data suggest that mast cell-mediated CysLTR2 signaling contributes to the development of interorgan visceral hypersensitivity. However, CysLTR2 is broadly expressed across multiple cell types including endothelial cells, immune cells and sensory neurons. To selectively silence CysLTR2 signaling in neurons innervating the bladder, we intramurally injected AAV9-Cre into the bladder wall of *Cysltr2*^*flox/flox*^ mice. Strikingly, viral-targeting of neurons for conditional deletion of *Cysltr2* mirrored the effects seen with pharmacological inhibition of CysLTR2, demonstrating significant attenuation of both bladder and colon hypersensitivity (**Extended Data Fig. 3c, d**). Collectively, these findings indicate that mast cells promote the bladder-colon hypersensitivity axis through CysLTR2 on sensory neurons.

In mice, it is increasingly appreciated that the GPCR MrgprB2 is a key sensor of mast cells^27^. In the skin, MrgprB2^+^ mast cells engage in bidirectional interactions with sensory neurons to mediate neurogenic inflammation and noxious sensations^28,29^. To evaluate the distribution of MrgprB2 expression in bladder cells, we used *MrgprB2*^*Cre*^*;Ai9*^*flox/flox*^ tdTomato reporter mice and found that approximately 96.3% of bladder mast cells were MrgprB2^+^ (**Extended Data Fig. 2d**). Spatially, we observed that MrgprB2 ^+^cells are uniquely enriched in the lamina propria (Fig. 3i **and Extended Data Fig. 2e**). These data indicated that mast cells in the bladder may have the capacity to sense irritants through MrgprB2 and subsequently activate nociceptors to trigger interorgan visceral hypersensitivity. To test this, we employed *MrgprB2*^−/−^ mice in the IC/BPS model. We found that bladder inflammation (Fig. 3j), urinary urgency (Fig. 3k), and bladder hypersensitivity (Fig. 3l) were all attenuated in the setting of MrgprB2 deficiency. Strikingly, deletion of this receptor alone caused a significant reduction in comorbid colon irritability (Fig. 3m) and dysmotility (Fig. 3n). Together, these findings demonstrate that mast cells and their associated MrgprB2 are key initiators of bladder-colon hypersensitivity.

## MRGPRX2 represents a therapeutic target in visceral hypersensitivity

**Figure 4:**
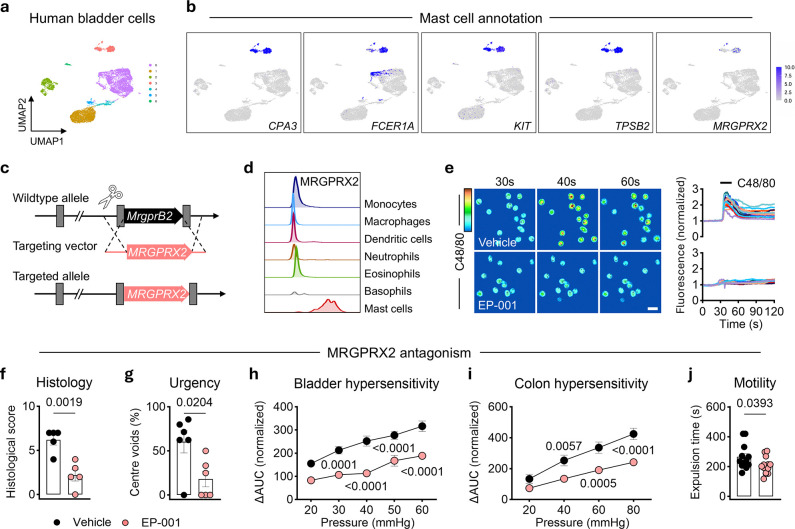
Pharmacological inhibition of MRGPRX2 alleviates interorgan hypersensitivity. **a**, Uniform Manifold Approximation and Projection (UMAP) of individual immune cell clusters in healthy human bladders (11203 cells from n = 3 human donors). **b**, Feature plots showing the expression of *MRGPRX2* genes in the canonical *CPA3, FCER1A, KIT*, and *TPSB2* expressing mast cell UMAP (924 mast cells from n=3 human donors). **c**, Schematic of humanized *huMRGPRX2*^*KI*^ mouse generation. **d**, Flow cytometric analysis of MRGPRX2 expression on indicated cell types in the bladder of the *huMRGPRX2*^*KI*^ mice. **e**, Left, representative Fluo-4 fluorescence heatmap images of *huMRGPRX2*^*KI*^ mouse peritoneal mast cells showing calcium influx induced by the administration of Compound 48/80 (10 μg ml^−1^) in the setting of vehicle or EP-001 (5 μg ml^−1^) pre-incubation. Scale bar, 20 μm. Right, representative imaging traces; normalized to baseline (see Methods). Each colored line represents an individual cell. **f**, **g**, **h**, **i**, **j**, Evaluations in bladder pathology (**f**; n = 5 per group), urinary urgency (**g**; n = 6 per group), bladder hypersensitivity (**h**; n = 10 and 8, respectively), colonic hypersensitivity (**i**; n = 13 and 14, respectively), and colonic motility (**j**; n = 12 and 14, respectively) in intravesically LL-37-challenged and vehicle-treated controls or EP-001-treated *huMRGPRX2*^*KI*^ mic. Data are presented as mean ± s.e.m. *P* values are shown in plots. **h**, **i**, Two-way repeated ANOVA with Sidak’s multiple comparisons test. **f**, **g**, **j**, Two-sided Student’s t-tests with Welch’s correction.

MRGPRX2 is considered the human ortholog of mouse MrgprB2^30^. Notably, the urinary bladder exhibits the second highest level of *MRGPRX2* expression among all surveyed tissues according to the Genotype-Tissue Expression (GTEx) portal (**Extended Data Fig. 4a**). To further identify the source of *MRGPRX2* expression in the bladder, we reanalyzed publicly available single-cell RNA sequencing datasets of the human bladder^31^ and confirmed that *MRGPRX2* was expressed by a cell cluster in the immune cell compartment (**Extended Data Fig. 4b**). We next identified mast cells based on the canonical markers *CPA3, FCER1A, KIT*, and *TPSB2* (Fig. 4a, b). Consistent with the above tracing results in *MrgprB2* reporter mice, *MRGPRX2* expression was exclusively observed in the mast cell cluster (Fig. 4b), indicating MRGPRX2 may have an evolutionarily conserved role, similarly to MrgprB2, in the human bladder. To manipulate MRGPRX2^+^ mast cells *in vivo*, we obtained a humanized MRGPRX2 knock-in mouse line (*huMRGPRX2*^*KI*^) wherein endogenous murine MrgprB2 was replaced with human MRGPRX2^32^ (Fig. 4c). Flow cytometric analysis of c-Kit^+^ CD200R3^+^ mast cells exhibited uniform expression of MRGPRX2, and all other immune cell lineages were devoid of MRGPRX2 expression in the bladder of *huMRGPRX2*^*KI*^ mice (Fig. 4d).

There are currently multiple MRGPRX2 antagonists in clinical development, but none are FDA-approved for any indication. Herein, we used a novel MRGPRX2 antagonist, EP-001, that has high potency activity against MRGPRX2 ligands on human mast cells. To assess the inhibition of MRGPRX2 by EP-001, we cultured peritoneal mast cells obtained from *huMRGPRX2*^*KI*^ mice to perform *in vitro* calcium imaging. Application of EP-001 blocked the calcium influx induced by the potent MRGPRX2 ligand Compound 48/80 (Fig. 4e). To further test its antagonism in a disease setting *in vivo*, we treated *huMRGPRX2*^*KI*^ mice with EP-001 upon IC/BPS induction. As expected, EP-001 administration effectively attenuated bladder inflammation (Fig. 4f), urinary urgency (Fig. 4g) and bladder hypersensitivity (Fig. 4h). Moreover, consistent with the above genetic studies in *MrgprB2*^−/−^ mice, EP-001-treated *huMRGPRX2*^*KI*^ mice also displayed reduced bladder perturbation-induced colonic irritability (Fig. 4i) and dysmotility (Fig. 4j). Collectively, these studies identify MRGPRX2 as a previously unrecognized therapeutic target for visceral hypersensitivity.

## Discussion

Classically, internal sensations such hunger, satiety, and sickness are mediated by vagal sensory neurons that reside in the nodose ganglia^33,34^. Beyond sensing somatic inputs, recent studies are shedding new light on the importance of spinal sensory neurons within the DRG in regulating a variety of visceral physiologies^35–37^. However, the spectrum of symptoms and the mechanisms underlying noxious sensations from the viscera remain poorly understood. Here, we identify a unique subpopulation of DRG sensory neurons that respond to cues from two distinct organs and critically mediate the development of interorgan visceral hypersensitivity. The existence of polyorganic sensory neurons may explain what drives multiorgan visceral pain syndromes, and why visceral pain is so difficult to localize.

Mast cells are strategically situated at barrier surfaces challenged by both external and internal threats. These are tissues that typically encounter and react to allergens along portals of entry such as the skin, airway, and upper gastrointestinal tract^38^. IgE acts on mast cells and is known to convert the antigen signal to defensive neural reflexes such as itching, coughing, and food avoidance^23,25,26,39,40^. Indeed, omalizumab (anti-IgE mAb) is now FDA-approved for urticaria, asthma, and food allergy. Although IgE reactivity is a hallmark of allergic responses, its blockade is not a treatment modality for visceral pain. Thus, the identification of MRGPRX2 as a critical pathway in bladder-colon irritation reveals an alternative mechanism by which mast cells trigger nociception and host-protective behavior in deeper and caudally distributed pelvic organs. We speculate that while IgE is evolutionarily designed to react to potential threats in organs that continuously sample the environment, MRGPRX2 may have particularly unique roles in organs with primarily expulsive functions such as the bladder.

While, classically, a sensorimotor reflex is thought to arise in response to a specific stimulus within a single organ^9^, visceral reflexes engage multiple organs to synchronize involuntary motor physiologies^41,42^. As early as in 1955, a bladder-colon reflex was observed in canines by Semba et al^16^, indicating how urination is coupled to defecation. Our current findings reveal how the sensory arc coordinates interorgan reflexes that may bypass traditionally assumed pathways of sensorimotor regulation. Given that autonomic motor neurons exhibit highly selective projection and organ-specific physiology^43^, we speculate that shared sensory networks establish a neuroanatomic basis for the timely coordination of visceral functions. Moreover, this interorgan neural circuit may be relevant across other mammalian organ systems.

Collectively, our studies uncover evolutionarily conserved mechanisms by which immune cells can convey signals from one organ to another in the absence of physical proximity via polyorganic sensory neurons. In the setting of inflammation, we unveil how homeostatic sensorimotor arcs can become hijacked to promote enhanced neural hypersensitivity to irritation, pain, and physiological dysfunction across multiple organ systems. These findings provide a molecular and cellular framework for the poorly understood syndrome of chronic pelvic pain. Importantly, targeting interorgan neuroimmune axes may represent novel therapeutic strategies for both inflammatory and sensory disorders.

## Methods

### Mouse lines

All animal experiments were approved by the Institutional Animal Care and Use Committee (IACUC) of the Icahn School of Medicine at Mount Sinai and performed in accordance with the guidelines of the National Institutes of Health, USA. Adult female mice were used for all experiments. Mice were provided with ad libitum access to a standard chow diet and sterile water and were housed in temperature- and humidity-controlled rooms with a 12hlight/dark cycle. All the mouse strains used in this study, except for *Cysltr2*^*flox*^ (MGI, 7289263), have been previously described: C57BL/6J (JAX, 000664), *Pirt*^*Cre*^ (JAX, 027214)^19^, Ai95D (*R26*^*RCL-GCaMP6F*^;JAX, 024105)^44^, *RC::FPDi* dual-recombinase responsive fluorescent/DREADD (*R26*^*FSF-mCherry-LSL-hM4Di*^;JAX, 029040)^20^, *Trpv1*^*Cre*^ (JAX,017769)^45^, Ai14 (*R26*^*LSL*-*tdTomato*^;JAX, 007914)^46^, *MrgprB2*^*Cre*^ (MGI, 574276)^30^, Ai9 (*R26*^*LSL-tdTomato*^;JAX, 007909)^46^, *MrgprB2*^*MUT*^ (MGI, 5749280)^30^, Mas-TRECK (MGI, 6407405)^22^, *huMRGPRX2*^*KI* 32^. All lines were kept on the C57BL/6J background.

### Murine IC/BPS model

Bladder instillation was performed under general anesthesia using isoflurane (induction: 3.0%; instillation: 1.0%) on a heating pad. Sterile saline or LL-37 (320 μM, AnaSpec AS-61302; Single letter amino acid sequence: LLGDFFRKSKEKIGKEFKRIVQRIKDFLRNLVPRTES) was administered intravesically to the bladder of 2–3-month-old female mice via transurethral catheterization, at a total volume of 100 μl with a dwell time of 1 hour as previously described^5^. Both saline and LL-37 were infused slowly to avoid distension and vesicoureteral reflux, and syringes were kept in place to prevent leakage during the procedure.

### Von Frey test

All experiments were conducted in a quiet behavioral room from 8–11 am each day. Mice were habituated in the testing environment for 2 days and were tested in a blinded manner. To test mechanical sensitivity, mice were placed in boxes on an elevated metal mesh floor, then their hind paws were stimulated with a series of von Frey monofilaments with logarithmically increasing force (0.02–2.56 g, Stoelting), applied perpendicularly to the plantar surface. Dixon’s up-down method was used to determine the 50% paw withdrawal threshold^47^.

To test pelvic mechanical sensitivity, mice were placed on the elevated wire grid as described above, then the suprapubic surface of their abdominal skin was stimulated with an innocuous 0.04 g von Frey filament 10 times at 1 min intervals. Positive responses were defined as a sharp retraction of the abdomen, immediate licking in the region of filament stimulation, and jumping during the stimulus.

### *In vivo* assessment of visceromotor response (VMR)

Abdominal electromyography (EMG) was used to monitor the VMR to urinary bladder distension (UBD) or colorectal distension (CRD) in lightly anaesthetized mice as previously described^48^.

#### Surgery for electrode transmitter implantation.

Surgical procedures were performed under general anesthesia using isoflurane (induction: 3.0%; maintenance: 1.0%) on a heating pad. Following sterilization, a 1.5–2.0 cm transverse incision was made on the lower right abdominal wall, and a 0.5 cm incision was made at the nape of the neck. The exposed ends of two Teflon-coated stainless-steel wires (Goodfellow, FE24-SW-000110) were sutured into the right obliquus externus abdominis and tunneled subcutaneously to exit at the base of the nape for future access. Both the abdominal and neck incisions were closed with a 6–0 non-absorbable suture (ETHILON, 697H). Mice were injected with an analgesic (Bupivacaine, 4 mg kg^−1^ i.d.) immediately after surgery. Animals were then housed individually and given a minimum of 2 days to recover prior to the experimental assay. On the day of VMR assessment, mice were lightly anesthetized using 0.5% isoflurane. The depth of anesthesia was confirmed that the flexion reflex response was present (evoked by pinching the paw), but that there was no righting reflex.

#### UBD.

A 24-gauge angiocatheter (BD, 381212), filled with approximately 500 μl of sterile saline, was gently introduced through the urethra and inserted into the bladder. The external urinary orifice was secured with a suture to prevent saline leakage during distension. The catheter was connected to a barostat (G&J Electronics, Distender Series II) for graded, pressure-controlled distension. Distensions were applied at 10, 20, 30, 40, 50, and 60 mmHg, with each distension lasting 10 sec and spaced at 5 min intervals, under computer control using ProtocolPlus Deluxe software.

#### CRD.

A lubricated custom balloon (1 cm in length) was tied to a PE50 catheter (Instech, 50–890-050) with silk 4.0 (FST, 18020–40) and gently inserted through the anus into the colorectum, positioned up to 2 cm past the anal verge. The catheter was secured to the base of the tail using tape to avoid any displacement during distension. The balloon catheter was connected to a barostat (G&J Electronics, Distender Series II) for graded and pressure-controlled balloon distension. Distensions were applied at 20, 40, 60, and 80 mmHg, each lasting 10 sec with 5 min intervals, under computer control using ProtocolPlus Deluxe software.

#### EMG signal acquisition and analysis.

A small piece of tinfoil was wrapped around the mouse tail with conductive adhesive and a ground wire was attached to the tinfoil. The abdominal electrodes were attached to the recording equipment of the signal acquisition. Signals from abdominal responses were amplified (World Precision Instruments, ISO-80) and recorded (LabChart software). The magnitude of the VMR at each distension pressure was quantified by calculating the area under the curve (AUC) during the 10 sec distension period, corrected for baseline activity (AUC pre-distension, 10 sec). VMRs were determined as the average of three to five pulses at each distension pressure.

### Spontaneous voiding assay

All experiments were conducted in a quiet behavioral room between 7–11 pm. Mice were put individually in home cages, with an absorbent acid-hardened filter paper (Whatman, grade 540) placed underneath. Mice were deprived of food and water and allowed to move freely for 4 hours, during which micturition events were captured as void spots on the paper. After testing, the paper was collected and imaged using a streaming camera (Logitech, Mevo Start) while illuminated by ultraviolet light on a transilluminator. The voiding patterns were quantified by another blinded experimenter. Images were then thresholded and converted to black and white (8-bit) using ImageJ/Fiji, and the total number of black pixels was counted and converted to square centimeters. Voids located within the center 50% of the total image area were defined as center voids, and voids smaller than 0.2 cm^2^ were classified as small voids. To ensure accurate analysis, spots smaller than 6.6 mm^2^ were excluded to eliminate potential noise that might be due to bite or claw marks, and fecal spots.

### Gut motility assay

All experiments were conducted in a quiet behavioral room between 8–11 am. Mice were kept in static disposable cages (Braintree) for 1 hour prior to the bead expulsion test to ensure that the distal colon was free of fecal pellets that could interfere with the entrance of a 3 mm diameter glass bead (Sigma, Z143928). Mice were lightly anesthetized with 1.0% isoflurane, and a glass bead was inserted 2 cm into the colon with a gavage cannula. The time for bead release was recorded. Only mice that recovered from anesthesia within 60 sec were included in the final quantification.

### Ussing Chamber

After acute colon dissection, the distal 2 cm of the colon was isolated for the assay. The muscularis and serosa layers were carefully removed under a dissecting microscope, and a piece of mucosa was mounted in an EasyMount Ussing chamber (Physiologic Instruments) using an insert with an aperture of 0.1 cm^2^ (Physiologic Instruments, P2303A). Reservoirs were filled with a standard Krebs solution containing 120 mM NaCl, 5.9 mM KCl, 14.4 mM NaHCO_3_, 1.35 mM NaH_2_PO_4_, 2.5 mM CaCl_2_, 1.2 mM MgCl_2_, and 11.5 mM glucose (pH 7.4). The solution was continuously bubbled with 5% CO_2_ and 95% O_2_ and maintained at 37°C using water jackets. *I*_SC_ was measured using an automatic voltage clamping device (Physiologic Instruments, EVC-4000) that compensates for the resistance of the solution between the potential-measuring Ag/AgCl electrodes that were kept in contact with the bathing solution via 3 M KCl-agar bridges. All experiments were conducted under a voltage clamp set at 0 mV. Tissues were equilibrated for 30 min to stabilize the *I*_SC_ before starting the experiment. Baseline electrical parameters were determined as the mean value over the 5 min immediately prior to Forskolin (10 μM) administration, which was applied to the luminal side to confirm tissue viability.

### Histology

#### Perfusion and post-fixation.

Mice were euthanized using CO_2_ and transcardially perfused with 10 ml cold 1× PBS (pH 7.4), followed by 10 ml cold 4% PFA. Bladders and distal colons were dissected out and fixed in 10% neutral buffered formalin (NBF, Sigma, HT501128) overnight at 4°C. Fixed tissues were sent to Histowiz (https://home.histowiz.com/) for H&E and toluidine blue staining and imaging by using a standard operating procedure and fully automated workflow. Tissues were preserved using paraffin embedding, and 4 μm central cross-sections of the bladder and colon were used for subsequent staining.

#### Histological severity score.

The severity of bladder inflammation was assessed using previously described methods^49^. Each slide was scored based on the following: thickness of the urothelial layer (0–2), extent of edema (0–4), and infiltration of polymorphonuclear leukocytes (0–3). The total histology score, ranging from 0–9, was calculated as the sum of each measure.

The histological score of the colon was assessed using previously described methods^50^. Slides were scored based on eight histological components: inflammatory infiltration, goblet cell loss, hyperplasia, crypt density, muscle thickness, submucosal infiltration, ulcerations and crypt abscesses, all categorized from 0–3. A total histological severity score, ranging from 0–24, was obtained by summing the eight item scores.

### Cholera toxin B (CTB) retrograde labelling

Surgical procedures were performed under general anesthesia using isoflurane (induction: 3.0%; instillation: 1.0%) on a heating pad. The pelvic organs were exposed following a 2–2.5 cm midline incision made on the lower abdominal wall. The bladder or distal colon were externalized and covered by a wet, sterile gauze during the microinjection. A total of 4–6 μl 0.1% CTB-647 (Invitrogen C34778) in PBS was slowly injected into the target organ, with 8–12 uniformly distributed injection sites (500 nl per site over 5 min) by using controlled microinjection pump (World Precision Instruments, Nanolitre 2000). CTB was intramurally delivered into the tissue layer between the mucosa and serosa, which is confirmed by the localized visible area without leakage. After injection, the abdominal wall and skin were sutured separately. Mice were injected with an analgesic (Bupivacaine, 4 mg kg^−1^ i.d.) immediately after surgery. Ganglia were collected 3–5 days after injection to allow the dye to reach the peripheral neuron soma.

### Whole-mount imaging of ganglia

#### Sample collection and preparation.

All ganglia of interest (DRG, NG and MPG) were dissected, fixed in 4% PFA and mounted in the refractive index matching solution (RIMS; 88%w/v Histodenz in 0.02 M PBS with 0.1% Tween-20,0.01% sodium azide, pH 7.5 with NaOH) with a silicone spacer on slides for confocal imaging.

#### Confocal imaging.

Mounted whole-mount ganglia were imaged with a LSM980 confocal microscope (ZEISS) using the following objectives: ×10/0.6NA, air immersion (ZEISS). Images were acquired using ZEN (ZEISS).

#### Quantification of CTB labelling in ganglia.

DRGs, NGs and MPG were taken from mice injected with CTB for whole-mount imaging as described above. T9–S3 DRGs, left/right NGs and MPG were quantified by another blinded experimenter for cell numbers labelled by CTB in 3D view using ZEN (ZEISS). The percentage of CTB double-positive cells in singly CTB-647 labelled cells was quantified.

### *In vivo* calcium imaging of L6 DRG neurons

#### Generation of *Pirt*^*Cre*^;*GCaMP6f*^flox/+^) mice.

All animal experiments were approved by the Institutional Animal Care and Use Committee (IACUC) at the University of Michigan and performed in accordance with the guidelines of the National Institutes of Health, USA. Heterozygous *Pirt*^*Cre*^ mice were bred with homozygous Ai95D mice to generate *Pirt*^*Cre*^*;GCaMP6f*^*flox*/+^ mice.

#### Surgery for DRG exposure and in vivo calcium imaging.

6-week-old female *Pirt*^*Cre*^*;GCaMP6f*^*flox*/+^ mice were used for *in vivo* calcium imaging of DRG. Mice were anesthetized with ketamine (100 mg kg^−1^) and xylazine (10 mg kg^−1^), then a laminectomy was performed from the L 6 to S 1 regions. The vertebral plate was drilled with a dental drill to create a window, and a transverse process was removed to expose the L6 DRG. Mice were placed on a heating pad to maintain a constant body temperature during surgery and imaging. During imaging, mice were placed on the microscope stage with spinal column clamps for stabilization to minimize movement and anesthesia was maintained using 1.5–2.0% isoflurane. A confocal laser scanning microscope (Olympus, FV3000) equipped with a 10×/0.4 NA air objective lens and a Piezo objective scanner (PI, P-725.CDD) was used to image the entire L6 DRG. Excitation/emission wavelengths of 488/500–540 nm were used to illuminate the green fluorescence of GCaMP6f. Time series images were acquired at a rate of one frame every 2.5 sec with an area of 512 × 512 pixels (z-axis with a thickness of 80 μm).

#### Urinary bladder and colorectal distension.

For urinary bladder distension, the bladder of anesthetized female mice was catheterized and connected to a saline-filled tubing linked to a sphygmomanometer. The external urinary orifice of mice was tied using a suture to prevent the leakage of saline during distension. For colorectal distension, a custom-made balloon catheter was inserted into the colon through the anus at a depth of 2 cm from the anus and the catheter was fixed to tails, then connected to a sphygmomanometer. Time series images were acquired at baseline and at increasing pressures of 20, 40 and 60 mmHg for urinary bladder distension or 40,60 and 80 mmHg for colorectal distension. Pressure was controlled using a sphygmomanometer and the duration of each distension was 30 sec at 3 min intervals.

#### Image processing and analysis.

Time series images were motion corrected using customized MATLAB scripts (Mathworks) and regions of interest (ROIs) on responding neurons were drawn manually using ImageJ/Fiji. Subsequently, the fluorescence dynamics of the ROIs were analyzed using custom MATLAB scripts. F_0_ was calculated as an average of 5 frames at baseline, then normalized GCaMP6f fluorescence was calculated as ΔF/F_0_ for each ROI. For background noise correction, signals from the regions surrounding the drawn somatic ROI were subtracted from the response of the somatic ROI^51^. Neurons were considered responsive if ΔF/F_0_ > 10%. Analysis was performed blinded to the treatment.

### Retrograde viral labelling of polyorganic sensory neurons

Microinjections were performed as described above. The following AAV9s were injected into visceral organs in *RC::FPDi* mice: Bladder AAV9-hSyn-Flpo-WPRE (OBiO H15027, 1.41 × 10^13^ VGs per ml, 5 μl) / AAV9-hSyn-mCherry-3xFLAG-WPRE (OBiO AOV063, 1.29 × 10^13^ VGs per ml, 5 μl), Colon AAV9-hSyn-Cre-WPRE (OBiO CN867, 3.83 × 10^13^ VGs per ml, 5 μl) / AAV9-hSyn-EGFP-3xFLAG-WPRE (OBiO AOV062, 2.43 × 10^13^ VGs per ml, 5 μl). For the following chemogenetic experiments, mice had at least 2 weeks of recovery period.

After recovering from retrograde viral labelling, *RC::FFPDi* mice were treated twice a day with intraperitoneal (i.p.) injections of deschloroclozapine (DCZ, 100 μg kg^−1^ in 100 μL of 1%DMSO/sterile saline; Tocris 7193) for 5 consecutive days followed by a 2-day washout prior to the behavioral and functional assays.

### Whole mount staining, clearing, and imaging of bladder

*Trpv1*^*Cre*^;Ai14^*flox/flox*^ or *MrgprB2*^*Cre*^;Ai9^*flox/flox*^ mice were terminally anaesthetized with isoflurane and transcardially perfused with 10 ml cold 1× PBS (pH 7.4) containing 20 U ml^−1^ heparin (Sigma-Aldrich, H3393), followed by 10 ml cold 4% PFA (Electron Microscopy Sciences, 1574100). Whole bladders were carefully dissected out and fixed further in 4% PFA overnight at 4°C. Fixed tissues were washed 3 times with PBS and stained following the standard SHIELD protocol (LifeCanvas). After delipidation procedures, tissues were passively labelled with fluorescein-conjugated Avidin (EMD Millipore, 189727, 1:500) in antibody blocking solution with 5% donkey serum at 37°C for 3 days. After index matching in Easylndex, the cleared tissues were embedded in a low melting point agarose (Thermo, 16520100) gel made of EasyIndex and imaged using the SmartSPIM light sheet microscope.

Whole-mount bladder images were rendered and analyzed using Imaris software. For the 3D rendering in Fig. 3b, different anatomical layers of the bladder were manually cropped based on the autofluorescence in the 488 channels. Next, tdTomato positive signals and Avidin positive signals were reconstructed using the Surface function. The shortest distances from the reconstructed Avidin to the reconstructed Trpv1 signals were quantified as the distance from mast cells to nociceptors in Fig. 3c. For Fig. 3i, tdTomato^+^Avidin^+^ cells and Avidin^+^ cells were manually counted and the ratio of tdTomato^+^Avidin^+^ to Avidin^+^ was used to calculate the percentage of MrgprB2 positive mast cells in different anatomical layers.

### Mast cell depletion

Targeted mast cell depletion was performed as previously described^22^. Briefly, Mas-TRECK and littermate (LM) control mice were treated daily with i.p. diphtheria toxin (DT, 250 ng in 100 μL of sterile saline; Sigma-Aldrich, D0564) for 5 consecutive days, mice had 1 weeks of recovery period before initiation of IC/BPS modeling. Depletion was verified by flow cytometry and Toluidine blue staining (**Extended Fig. 2a-c**).

### Flow cytometry

#### Tissue digestion.

Mouse bladders were digested as previously described^52^. In brief, bladders were collected in cold HBSS and minced, then transferred to 1 ml of freshly prepared digestion solution (0.06 mg ml^−1^ Liberase TM (Roche, 5401127001), 0.125mg ml^−1^ DNAse I (Roche, 10104159001) in HBSS), and incubated for 1 hour at 37°C in a water bath with vigorous shaking at 15 min intervals until a glassy appearance was reached. Reactions were stopped by adding cold stop buffer (2% FBS, 0.2 mM EDTA in PBS). Cells were filtered using a 100 μm filter (Fisher Scientific, 22–363-549), collected by centrifugation, and resuspended in staining buffer (2% FBS, 5 mM EDTA in PBS).

#### Cell staining.

Single-cell suspensions were stained with the Zombie NIR^™^ Fixable Viability Kit (BioLegend, 423106, 1:500) followed by treatment with anti-CD16/32 (Fc Block, clone 2.4G2, BioXCell, BE0307, 1:50) and cell-surface staining with specific antibodies in PBS. The following antibodies were used at a dilution of 1:300 except where otherwise indicated: BV605-c-Kit (BioLegend, 135122, clone ACK2); Alexa Fluor 532-CD45 (eBioscience, 58–0451-82, clone 30-F11); PerCP/Cy5.5-Ly6G (BioLegend, 127616, clone 1A8); BV711-Ly6G (BioLegend, 127643, clone 1A8); PerCP/Cy5.5-CD19 (eBioscience, 45–0193-82, clone eBio1D3 (1D3)); PerCP/Cy5.5-CD5 (eBioscience, 45–0051-82, clone 53–7.3); PerCP/Cy5.5-CD3e (eBioscience, 45–0031-82, clone 145–2C11); PerCP/Cy5.5-CD11b (eBioscience, 45–0112-82, clone M1/70); BV510-CD11b (BioLegend, 101245, clone M1/70); PerCP/Cy5.5-NK1.1 (eBioscience, 45–5941-82, clone PK136); PE/Cy7-CD200R3 (BioLegend, 142212, clone Ba13); APC-CD49b (BioLegend, 108910, clone DX5); PE/Dazzle 594-CD49b (BioLegend, 108924, clone DX5); BUV395-CD11c (BD Horizon, 564080, clone HL3); BUV496-F4/80 (BD OptiBuild, 750644, clone T45–2342); BV421-MHCII (BioLegend, 107631, clone M5/114.15.2); BV570-Ly6c (BioLegend, 128030, clone HK1.4); PE-SiglecF (BioLegend, 155506, clone S17007L); APC-MRGX2 (BioLegend, 359005, clone K125H4). Cells were fixed with BD Cytofix/Cytoperm^™^ Fixation and Permeabilization Solution (BD, 554714).

#### Flow cytometric analysis.

Flow cytometry was carried out using a Cytek Aurora (CYTEK) flow cytometer equipped with the following lasers: 355 nm (ultraviolet), 405 nm (violet), 488 nm (blue), 561 nm (yellow green), and 640 nm (red). Data were analysed using FlowJo software v10.8.1.

#### Gating strategy.

Mast cell gating: FSC-A vs. FSC-H to exclude doublets, live CD45^+^FSC-W^low^SSC-W^low^CD3e^−^CD5^−^CD19^−^NK1.1^−^Ly6G^−^CD11b^−^c-Kit^+^CD200R3^+^ ; Dendritic cell gating: FSC-A vs. FSC-H to exclude doublets, live CD45^+^FSC-W^low^SSC-W^low^MHCII^+^F4/80^−^CD11c^+^; Macrophage gating: FSC-A vs. FSC-H to exclude doublets, live CD45^+^FSC-W^low^SSC-W^low^MHCII^+^c-Kit^−^F4/80^+^; Monocyte gating: FSC-A vs. FSC-H to exclude doublets, live CD45^+^FSC-W^low^SSC-W^low^F4/80^−^CD11b^+^Ly 6c^+^; Neutrophil gating: FSC-A vs. FSC-H to exclude doublets, live CD45^+^FSC-W^low^SSC-W^low^MHCII^−^-CD11b^+^Ly6G^+^; Eosinophil gating: FSC-A vs. FSC-H to exclude doublets, live CD45^+^FSC-W^low^SSC-W^low^MHCII^−^SiglecF^+^CD11b^+^; Basophil gating: FSC-A vs. FSC-H to exclude doublets, live CD45^+^FSC-W^low^SSC-W^low^CD3e^−^CD5^−^CD19^−^NK1.1^−^CD11c^−^ c-Kit^−^CD49b^+^CD200R3^+^.

### CysLTR2 antagonist treatment

After IC/BPS model establishment, mice were intraperitoneally administered with vehicle or CysLTR2 antagonist HAMI3379 (1 mg kg^−1^ in 1× PBS; Cayman, 10580) daily for 7 consecutive days before the functional assays.

### *In vitro* calcium imaging of peritoneal mast cells

2–3-month-old *huMRGPRX2*^*KI*^ female mice were euthanized by CO_2_. Peritoneal lavages were made using an intraperitoneal injection of 7 ml cold mast cell dissociation media (MCDM; HBSS with 3% fetal bovine serum and 10 mM HEPES, pH 7.2), and cells were spun down at 200*g* for 10 min. The pellet from each mouse was resuspended in 2 ml MCDM, layered over 4 ml of an isotonic 70% Percoll suspension (2.8 ml Percoll, 320 μl 10× HBSS, 40 μl 1 M HEPES, 830 μl MCDM), and spun down at 500*g* for 20 min at 4°C. Mast cells were resuspended at 1×10^4^ – 5×10^4^ cells ml^−1^ in DMEM with 10% fetal bovine serum and 25 ng ml^−1^ recombinant mouse stem cell factor (Sigma, S9915), and plated onto glass coverslips coated with 0.1 mg ml^−1^ Poly-D-Lysine (Gibco, A38904–01). After 2 hours of incubation at 37°C with 5% CO_2_, mast cells were loaded with Fluo-4 (Invitrogen, F10471) for 30 min at 37°C. The cells were then transferred to DMEM containing 10% fetal bovine serum with vehicle or 5 μg ml^−1^ EP-001 for another 30 min at 37°C. After EP-001 incubation, cells were immediately used for calcium imaging. Fluorescence was recorded for 30 sec for a baseline measurement, followed by treatment with 10 μg ml^−1^ Compound 48/80 (Sigma, C2313) for 2 min, then treated with 100 μM ATP (Tocris, 3209) as a positive control to confirm cell viability. The fluorescent intensity was normalized to baseline (F/F_0_) to generate the traces.

### MRGPRX2 antagonist treatment

After IC/BPS model establishment, *huMRGPRX2*^*KI*^ mice were orally gavaged with vehicle or EP-001 (50 mg kg^−1^ in 100 μL of 5% DMSO/5% Solutol/90%1× PBS; Escient Pharmaceuticals) twice a day for 7 consecutive days before the behavioral and functional assays.

### Single-cell RNA sequencing analysis

Human bladder data was obtained from CZ BioHub Tabula Sapiens (GEO Accession ID: GSE201333)^31^. Human bladder data was preprocessed to remove dead cells, multiplets, or ambient RNA contamination^31^. All the data was initially grouped based on compartments (endothelial, epithelial, immune, or stromal) to identify the expression of MRGPRX2 across the entire bladder. Immune cell data was subsequently processed using the standard Seurat pipeline. Immune cells were projected into their own UMAP space and clustered using the FindNeighbors() and FindClusters() functions in R Studio. Mast cells were identified based on the expression of canonical markers including *CPA3, KIT, FCER1A*, and *TPSB2*.

### GTEx data analysis

Bulk RNA-Seq data from various human organs and the associated metadata was downloaded from the GTEX Portal (https://gtexportal.org/home/). The expression levels of *MRGPRX2* were converted from Transcripts Per Million (TPM) to log_10_[TPM+1]. Samples from the “Cells - EBV-transformed lymphocytes” and “Cells - Cultured fibroblasts” were excluded from the analysis. The remaining data were then grouped by the “SMTS” classification and sorted based on highest to lowest expression levels.

### Statistics, blinding and randomization and reproducibility

Statistical analyses were performed in GraphPad Prism software (v.9.5.0). All statistical tests are described in the corresponding figure legends. Prior to analysis, all data was first analyzed for normal distribution using Kolmogorov-Smirnov or Shapiro-Wilk tests. Unless otherwise stated, data are presented as mean ± s.e.m. Differences were considered statistically significant at *P* < 0.05. The sample size for animals or cells used is indicated in the figure legends. Genetically modified mice and control littermates were randomly allocated from different cages of female mice for analyses. Group sizes were determined to ensure adequate statistical power for all measurements, with genotype assignments made blind until after data collection. Randomization was applied to all experiments comparing drug versus vehicle treatments.

All *in vivo* experiments were performed at least twice or grouped from two independent cohorts with the same conclusion. For representative images, the numbers of independently experimental repetitions with similar results were as follows: Figs 1c–i (two), 2b (three), 2 e (four), 3a,b (four), 4d (three), 4e (two) and Extended Data Figs. 1a (three), 1d (four), 2a–c (three), 2d (three), 2e (four).
